# QTL Mapping of SPAD Values Associated with Leaf Color in Bunching Onion

**DOI:** 10.3390/genes17050534

**Published:** 2026-04-30

**Authors:** Tetsuya Nakajima, Kouei Fujii, Kenji Watanabe, Yoichi Mizukami, Masaru Bamba, Shusei Sato, Masayoshi Shigyo

**Affiliations:** 1 Laboratory of Vegetable Crop Science, Division of Life Science, Graduate School of Sciences and Technology for Innovation, Yamaguchi University, Yamaguchi City 753-8511, Yamaguchi, Japan; 2 Yamaguchi Prefectural Agriculture and Forestry General Technology Center, 10318 Mure, Hofu City 747-0004, Yamaguchi, Japan; 3 Center For Gene Research, Yamaguchi University, 1-1-1 Minamikogushi, Ube City 755-8505, Yamaguchi, Japan; 4 Graduate School of Life Sciences, Tohoku University, 2-1-1 Katahira, Aoba-ku, Sendai City 980-8577, Miyagi, Japan

**Keywords:** *Allium fistulosum*, SPAD, QTL, linkage map

## Abstract

**Background/Objectives**: The dark green leaf color trait in bunching onion (*Allium fistulosum* L.) is an important agronomic trait closely associated with market value; however, its genetic basis remains poorly understood. This study aimed to identify quantitative trait loci (QTLs) associated with leaf color using SPAD values as a phenotypic indicator. **Methods**: An $F_{2}$ population derived from a cross between the dark green line YSG1go and the light green line Asagikei-KUJYO was used. A linkage map was constructed based on RNA-seq-derived SNP markers, and SPAD values were measured for QTL analysis. **Results**: The linkage map consisted of eight linkage groups with a total length of 2103.0 cM and 765 mapped markers. SPAD values showed significant differences between the parental lines, with high broad-sense heritability ($H^{2}$ = 0.76), indicating a strong genetic contribution to this trait. Multiple significant QTLs were detected on chromosomes 4 and 5, each explaining 27.4–38.1% of the phenotypic variance. The direction of allelic effects differed among QTLs, suggesting that favorable alleles are distributed between the parental lines. In addition, genes related to chloroplast protein translation were identified within the QTL regions. **Conclusions**: SPAD values are a suitable indicator for genetic analysis of leaf color in bunching onion, and the QTLs identified in this study provide valuable information for molecular breeding aimed at improving dark green leaf color.

## 1. Introduction

Bunching onion (*Allium fistulosum* L.), also known as Welsh onion, green onion, spring onion, or scallion, is widely distributed from Siberia to tropical Asia. In East Asia, numerous cultivars adapted to diverse environmental conditions have been developed [1,2]. Bunching onion is a leafy vegetable in which the leaves are consumed and is available throughout the year. Its vibrant green color and characteristic flavor are important quality attributes in various dishes [3,4,5], and the dark green phenotype is a key agronomic trait closely associated with market value.

Leaf color is primarily determined by chlorophyll content and is closely associated with photosynthetic capacity. Chlorophyll is a key pigment in photosynthesis and plays a central role in light absorption and electron transport in photosystem II (PSII). PSII is highly sensitive to light, and its function declines under high light conditions due to photoinhibition [6]. Therefore, the balance between photodamage and repair is critical for maintaining photosynthetic activity [7], and may influence chlorophyll content and leaf color.

SPAD values are widely used for the quantitative evaluation of leaf color, as they provide a simple and non-destructive estimate of leaf chlorophyll content. SPAD values have been reported to be strongly correlated with chlorophyll concentration [8] and thus serve as an effective indicator of leaf greenness. Furthermore, SPAD values have been widely applied as indicators of plant nutritional status and photosynthetic performance in a wide range of crops, including major cereals such as rice (*Oryza sativa* L.) and wheat (*Triticum aestivum* L.), as well as vegetable crops such as onion (*A. cepa* L.) [9,10,11].

In recent years, molecular breeding based on genomic information has advanced rapidly. The whole-genome sequence of bunching onion was reported in 2022, providing a foundation for gene-level trait analysis [12,13]. For the analysis of quantitative traits, genome-wide association studies (GWAS) have been widely applied in major crops such as rice and maize (*Zea mays* L.), supported by the availability of genomic resources and genetically diverse populations [14,15]. In contrast, genomic resources for bunching onion remain limited, and quantitative trait locus (QTL) analysis is still the primary approach. Indeed, QTL analyses have been reported for various traits in bunching onion, including morphological traits, pseudostem pigmentation, seedling growth, and pungency, indicating that these traits are controlled by multiple genetic loci [16,17,18]. However, the genetic basis of leaf color remains poorly understood.

In this study, a genetic linkage map was constructed using markers obtained by mRNA-based genotyping-by-sequencing (mRNA-GBS) in an $F_{2}$ population derived from bunching onion lines exhibiting dark green and green phenotypes. QTL analysis was performed using SPAD values as a phenotypic indicator to identify regions associated with variation in this trait. Furthermore, candidate genes located within the detected QTL regions were investigated to gain insights into the genetic basis of the dark green phenotype. This study provides fundamental knowledge for the development of molecular markers associated with leaf color and is expected to contribute to efficient marker-assisted breeding.

## 2. Materials and Methods

### 2.1. Plant Materials and Growth Conditions

In this study, two bunching onion lines were used as parental materials: ‘YSG1go’ (YSG1), a dark green fixed line derived from Yamaguchi Prefecture, and ‘Asagikei-KUJYO’ (KUJYO), a green line. KUJYO was used as the seed parent and YSG1 as the pollen parent to generate $F_{1}$ plants. The $F_{1}$ plants were subsequently self-pollinated to produce an $F_{2}$ segregating population.

Seeds obtained from these crosses were sown on 12 May 2021 in a greenhouse at the Yamaguchi Prefectural Agriculture and Forestry General Technology Center (34° N, 131° E), where all plants were grown under greenhouse conditions. YSG1, KUJYO, and $F_{1}$ plants were harvested on 13 July 2021, while the $F_{2}$ population was harvested on 16 and 19 July 2021.

Plants were cultivated on ridges 90 cm in width with six rows per ridge. The spacing between rows was 12 cm, and the sowing density was 120 seeds/m^2^. The total nitrogen application rate was 1.0 kg/a, with 0.5 kg/a applied as basal fertilizer at sowing and 0.5 kg/a applied as topdressing at the two-leaf stage.

The irrigation regime was managed according to plant developmental stages. On the day of sowing, 48 L/m^2^ of water was applied, and soil water tension was adjusted to pF 1.5. During the germination period (0–4 days), irrigation was applied at 24 L/m^2^ per event to maintain soil moisture within a pF range of 1.5–1.6. In the cotyledon stage (4–11 days), water was supplied every 2–3 days at 6 L/m^2^, maintaining pF values between 1.6 and 1.8. At the one-leaf stage (11–18 days), irrigation was conducted every 2–3 days with 10 L/m^2^ of water, keeping soil moisture within a pF range of 1.7–2.0. During the two-leaf stage (18–28 days), irrigation frequency was increased to daily application at 12 L/m^2^, maintaining pF values between 1.6 and 1.8. At the three-leaf stage (28–38 days), irrigation was reduced to every three days at 6 L/m^2^, with soil water tension maintained at pF 2.0–2.3. During the four-leaf stage (38–49 days), irrigation was performed almost daily at 12 L/m^2^ to maintain pF values between 1.8 and 2.0. At the five-leaf stage (49–59 days), irrigation was applied every three days at 5 L/m^2^, maintaining soil water tension within a pF range of 2.0–2.5. From the six-leaf stage (59 days onward), irrigation was adjusted according to soil moisture conditions.

Meteorological data for Yamaguchi City (34° N, 131° E), including air temperature (average, maximum, and minimum), relative humidity (average and minimum), and sunshine duration from 12 May to 19 July 2021, were obtained from the Japan Meteorological Agency (https://www.data.jma.go.jp/stats/etrn/index.php, accessed on 31 March 2026) (Supplementary Table S1).

### 2.2. Sampling and SPAD Measurement

Ten individuals were randomly selected from each of KUJYO, YSG1, and the $F_{1}$ population, while 79 individuals from the $F_{2}$ segregating population were used for analysis. Prior to measurement, the surface wax was removed using a commercially available neutral kitchen detergent. SPAD values were measured using a chlorophyll meter (SPAD-502Plus, KONICA MINOLTA, INC., Tokyo, Japan) and calculated as the average of readings taken at the central portions of the first and second leaves.

### 2.3. Sample Preparation

After SPAD measurement, the samples were immediately frozen at −80 °C. The frozen samples were then freeze-dried for 3 days using a freeze dryer equipped with a vacuum pump (VD-250R, TAITEC, Saitama, Japan). The freeze-dried samples were subsequently ground into a fine powder using a small blender.

### 2.4. Determination of Pigment Compounds

Exactly 20 mg of freeze-dried leaf tissue was weighed and transferred into a 15 mL tube, followed by the addition of 2.5 mL of chilled acetone. The mixture was vortexed for 2 min and then subjected to ultrasonication for 20 min under low-temperature conditions. After centrifugation at 10 °C and 5000 rpm for 5 min, the supernatant was collected. The residue was re-extracted with an additional 2.5 mL of chilled acetone using the same procedure, and the supernatants were combined. All collected supernatants were filtered through a 0.45 μm membrane filter (Advantec, Tokyo, Japan) and used for the analysis of pigment compounds, including chlorophyll *a*, chlorophyll *b*, lutein, and β-carotene. The sample solutions were stored at −20 °C and analyzed within 3 days.

Pigment compounds were quantified using an HPLC system (L-7000 series, HITACHI, Tokyo, Japan) equipped with a UV–Vis detector (L7420, HITACHI, Tokyo, Japan) at a detection wavelength of 435 nm. Separation was performed using a LiChroCART 250-4.0 Lichrospher 100 RP-18 column (5 μm; KANTO CHEMICAL, Tokyo, Japan). The mobile phase consisted of two solvents: (A) 80% methanol solution (prepared by mixing 400 mL of HPLC-grade methanol, 50 mL of ultrapure water, and 50 mL of 100 μM HEPES buffer, pH 7.5) and (B) ethyl acetate.

The gradient elution program was as follows: (i) initial condition, 100% A; (ii) a linear gradient to 50% A and 50% B over 20 min; and (iii) an isocratic hold at 50% A and 50% B for 30 min. The flow rate was 1.0 mL/min, the column temperature was maintained at 30 °C, and the injection volume was 50 μL. The concentrations of each pigment were calculated based on calibration curves prepared using standard compounds.

### 2.5. Transcriptome Sequencing

RNA sequencing data acquisition for each sample and construction of the unigene dataset were performed according to previously reported methods [19]. Total RNA was extracted from leaf tissues of one individual from each of KUJYO and YSG1, and 79 individuals from the $F_{2}$ population, using the RNeasy Plant Mini Kit (QIAGEN Sciences, Germantown, MD, USA). RNA quality was assessed using an Agilent 2100 Bioanalyzer (Agilent Technologies, Palo Alto, CA, USA), and samples with an RNA integrity number (RIN) greater than 8.0 were used for subsequent analyses.

cDNA libraries were prepared using the ${\mathrm{TruSeq}}^{\mathrm{TM}}$ RNA Sample Preparation Kit (Illumina, San Diego, CA, USA) according to the manufacturer’s instructions. Sequencing was performed on an Illumina HiSeq 2500 platform (Illumina, San Diego, CA, USA).

### 2.6. SNP Detection and Selection

RNA sequencing reads were filtered using PRINSEQ v0.20.4 [20] and the fastx_clipper tool from the FASTX-Toolkit (http://hannonlab.cshl.edu/fastx_toolkit/, accessed on 31 March 2026). The filtered single-end reads were mapped to the KUJYO unigene sequences using Bowtie v2.1.0 [21] in end-to-end mode. The resulting sequence alignment/map (SAM) files were converted to BAM format using SAMtools v0.1.19 [22].

SNP calling was performed using the mpileup function in SAMtools v0.1.19 and the mpileup2snp function in VarScan v2.3, and variant call format files containing SNP information were generated.

### 2.7. SNP Marker Filtering and Selection

mRNA-derived SNP markers were consolidated to reduce redundancy among SNPs located within the same unigene based on genotype patterns of the parental lines and $F_{2}$ individuals. Specifically, SNPs with a genotype concordance rate of ≥95% (excluding missing values) were grouped into the same cluster, and the largest cluster within each unigene was selected as representative. For SNPs within the representative cluster, genotypes for each individual were integrated by majority voting to generate a consensus marker.

Furthermore, markers showing homozygous polymorphism between the parents (A/B type; A represents the KUJYO allele and B represents the YSG1 allele) were retained. For markers in which one parent was heterozygous (H), only those that showed segregation in the $F_{2}$ population were retained for further analysis, whereas markers showing no segregation were excluded.

Based on the quality assessment of RNA-seq data, one individual was excluded due to insufficient data quality. Consequently, a total of 78 individuals were used for linkage map construction and QTL analysis.

### 2.8. Linkage Analysis

Linkage analysis was performed using JoinMap 5 (Kyazma B.V., Wageningen, The Netherlands) under the $F_{2}$ population model based on the selected markers and SNP genotype data from 78 individuals. Prior to analysis, markers with more than 10 missing values and those showing severe segregation distortion ($\chi^{2}$ test, $p<1.0\times 10^{-6}$) were excluded to improve mapping accuracy. In addition, markers with identical genotypes were removed to reduce redundancy. Markers exhibiting excessively large genetic distances and lacking appropriate linkage relationships with other markers were also excluded.

Marker grouping was conducted using SNP data from 78 $F_{2}$ individuals at a LOD threshold of 10. The resulting linkage groups were assigned to chromosomes based on reference genome information from the Allium TDB database (https://alliumtdb.kazusa.or.jp/, accessed on 31 March 2026). Subsequently, linkage analysis was repeated for each linkage group at a LOD threshold of 5 to construct the final linkage map.

### 2.9. QTL Analysis

QTL analysis was performed using MapQTL 7 (Kyazma B.V., Wageningen, The Netherlands). SPAD values were used as phenotypic data, and interval mapping was conducted based on the constructed linkage map. The presence of QTLs was determined based on LOD scores, and regions showing significant LOD peaks were identified as QTLs. Significance thresholds for LOD scores were determined by permutation tests (1000 permutations) implemented in MapQTL. Genome-wide LOD thresholds corresponding to the 5% and 1% significance levels were used to declare significant QTLs. The confidence interval for each QTL was defined as the 1.5-LOD support interval around the LOD peak.

### 2.10. Candidate Gene Identification

Candidate genes within the QTL intervals were identified based on reference genome annotation obtained from the Allium TDB database (Kazusa DNA Research Institute). The genetic positions of QTLs were converted to physical positions using the same database.

### 2.11. Identification of Amino Acid Substitutions

Coding sequences were determined by identifying start codons from the nucleotide sequences. SNP information was then used to infer amino acid sequences, which were compared between the parental lines to identify amino acid substitutions.

### 2.12. Correlation Analysis Between Gene Expression and SPAD Values

To examine whether transcript abundance of selected candidate genes (*EF-G* and *GUF1*) was associated with SPAD values, RNA-seq-derived gene count data from the $F_{2}$ population were analyzed. Raw counts were normalized to counts per million (CPM), and $\log_{2}$(CPM + 1) values were used for analysis. Pearson’s correlation coefficients were calculated between gene expression levels and SPAD values.

## 3. Results

### 3.1. Phenotypic Variation in SPAD Values and Pigment Traits in the $F_{2}$ Population

**Figure 1:**
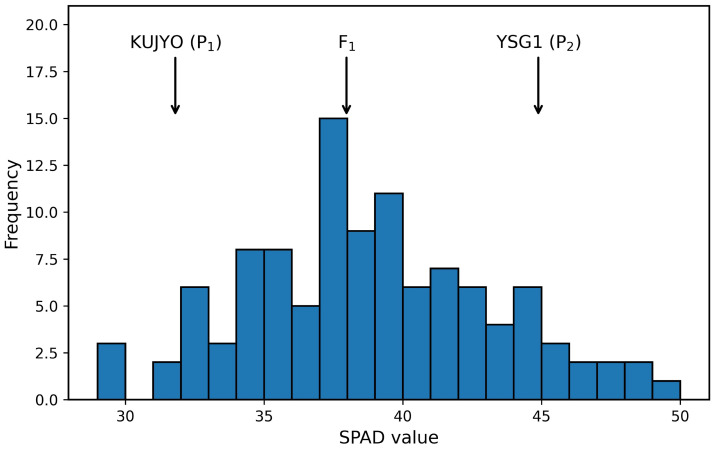
Frequency distribution of SPAD values in the $F_{2}$ population with mean values of the parental lines and $F_{1}$.

The measured values of SPAD and pigment compounds are presented in Supplementary Table S2. The mean SPAD value of YSG1 was significantly higher than that of KUJYO at the 1% significance level, and the broad-sense heritability ($H^{2}$) was estimated to be high (0.76) (Table 1). The mean SPAD value of the $F_{1}$ population was intermediate between the two parental lines. In the $F_{2}$ population, SPAD values showed a continuous distribution (Figure 1).

Similarly, for other pigment compounds, YSG1 exhibited significantly higher values than KUJYO. In contrast, the broad-sense heritability of these traits was estimated to be negative and was therefore treated as zero (Supplementary Table S3).

### 3.2. SNP Filtering and Linkage Map Construction

A total of 31,248 SNP markers were initially detected from the RNA-seq data. After applying filtering and selection criteria, 6388 high-quality SNP markers were retained for subsequent analyses. Of these, 765 markers were ultimately used for linkage map construction following linkage analysis with JoinMap 5 and subsequent refinement to improve map quality. The markers used for linkage map construction are listed in Supplementary Table S4.

**Figure 2:**
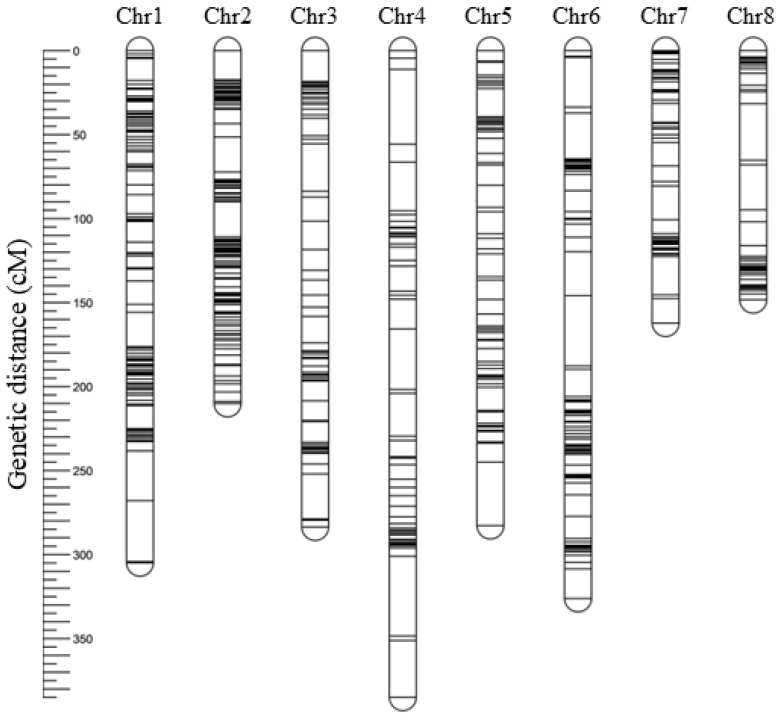
Genetic linkage map constructed for the $F_{2}$ population.

The resulting linkage map consisted of eight linkage groups, with a total map length of 2103.0 cM (Figure 2, Table 2). The average marker interval was approximately 2.78 cM, and the maximum gap was 47.5 cM.

### 3.3. QTL Analysis for SPAD Values

**Figure 3:**
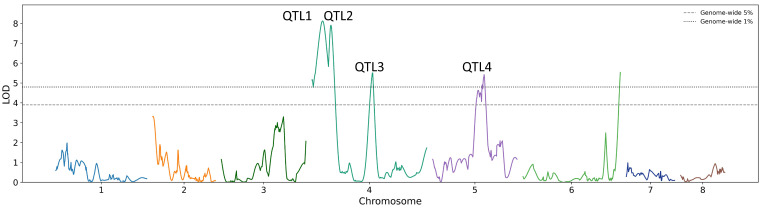
QTL Each chromosome is represented by a different color. analysis of SPAD values across the linkage groups. Horizontal dashed lines indicate the genome-wide significance thresholds at the 1% and 5% levels determined by permutation tests (n = 1000). Each chromosome is represented by a different color.

QTL analysis for SPAD values identified three significant QTLs on chromosome 4 at the 1% significance level and one QTL on chromosome 5 at the 5% significance level (Figure 3, Table 3). A signal exceeding the 1% threshold was also detected on chromosome 6; however, the peak was located at the terminal region of the linkage group and was therefore excluded from further analysis.

QTL1 was located at approximately 36.14 cM on chromosome 4, with a LOD score of 8.12, explaining 38.1% of the phenotypic variance. The 1.5-LOD support interval ranged from 20.14 to 50.14 cM; however, the corresponding physical position could not be clearly determined due to the absence of mapped markers in this region. QTL2 was located at approximately 63.67 cM on chromosome 4, with a LOD score of 7.91, explaining 37.3% of the phenotypic variance. The 1.5-LOD support interval ranged from 57.67 to 71.45 cM, corresponding to a physical position of 1133.8 Mb. Based on the annotation of markers within this region, a gene annotated as Translation factor GUF1 homolog, chloroplastic (*GUF1*) was identified as a candidate gene. QTL3 was located at approximately 201.69 cM on chromosome 4, with a LOD score of 5.51, explaining 27.8% of the phenotypic variance. The 1.5-LOD support interval ranged from 194.66 to 208.01 cM, corresponding to a physical interval of 827.6–837.9 Mb. In this region, genes with unknown functions were predominantly identified based on marker annotation. QTL4 was located at approximately 172.52 cM on chromosome 5, with a LOD score of 5.42, explaining 27.4% of the phenotypic variance. The 1.5-LOD support interval ranged from 145.61 to 177.35 cM, corresponding to a physical interval of 114.8–636.5 Mb. This region included genes annotated as elongation factor G (*EF-G*), PI4 kinase, UDP-galactose transporter, and transcription-related proteins based on marker annotation.

Based on the additive effects of each QTL, the parental alleles contributing to increased SPAD values differed among loci. Specifically, the KUJYO-derived allele contributed to increased SPAD values at QTL1 and QTL2, whereas the YSG1-derived alleles contributed to increased SPAD values at QTL3 and QTL4.

**Figure 4:**
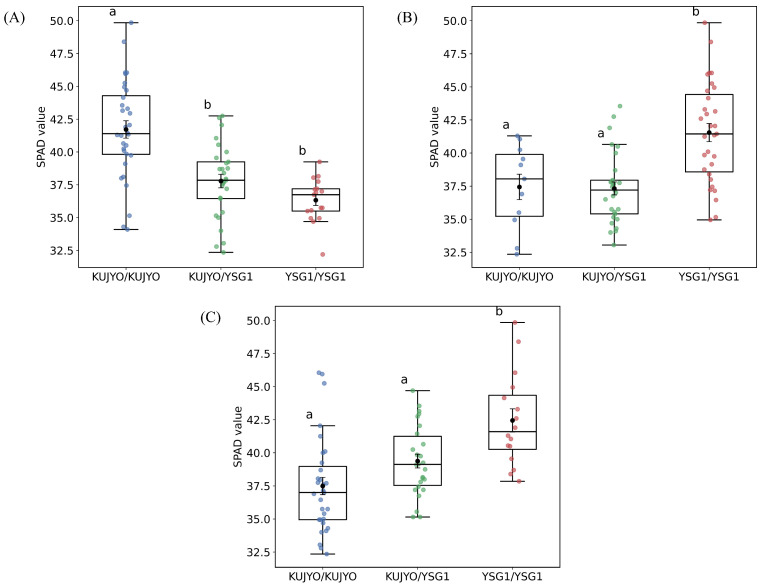
Effects of genotype at the peak markers for each QTL on SPAD values. Boxplots show the distribution of SPAD values for each genotype at QTL2 (**A**), QTL3 (**B**), and QTL4 (**C**). Different letters indicate significant differences among genotypes (Tukey’s test, p<0.05).

Comparison of SPAD values among genotypes at the peak markers in the $F_{2}$ population revealed that the mean SPAD value was significantly higher in homozygotes for the KUJYO allele at QTL2 (Figure 4A), whereas it was significantly higher in homozygotes for the YSG1 allele at QTL3 and QTL4 (Figure 4B,C).

To further examine the relationship between the genetic linkage map and the physical map, the physical positions of markers within each QTL region were compared (Supplementary Table S5). The degree of consistency between genetic and physical positions varied among QTLs. Markers within the QTL3 region showed relatively consistent ordering between the genetic and physical maps. In contrast, markers within the QTL1 and QTL2 regions were distributed across a relatively broad physical interval (approximately 900–1200 Mb) and exhibited local inconsistencies in marker order. Furthermore, markers within the QTL4 region showed more pronounced variation in physical positions, including large positional shifts.

**Figure 5:**
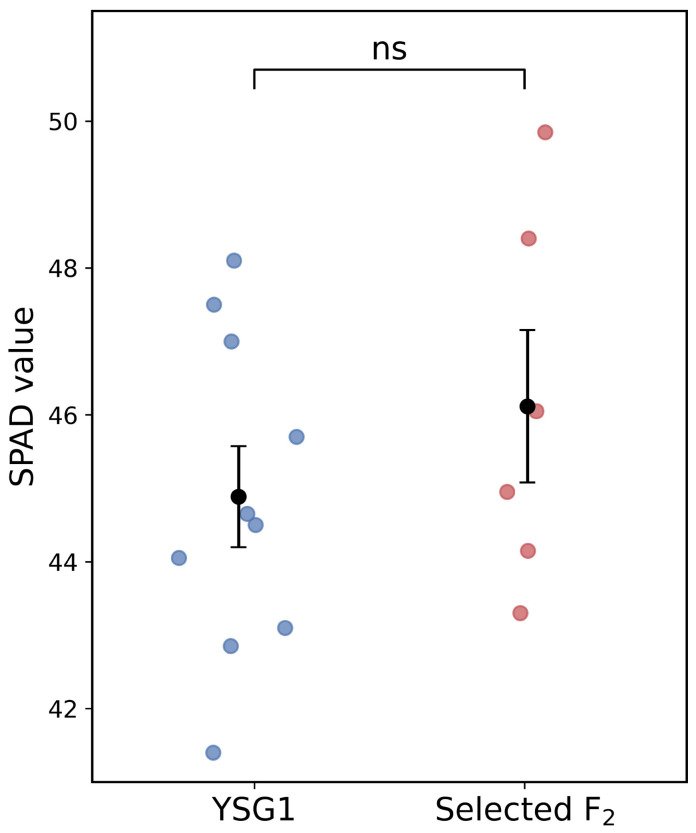
Comparison of SPAD values between selected $F_{2}$ individuals carrying allele combinations that increase SPAD values at QTL2, QTL3, and QTL4 and the parental line YSG1. Each point represents an individual. Black circles indicate the mean ± SE. Differences between groups were not significant (ns, p>0.05).

Furthermore, $F_{2}$ individuals carrying combinations of favorable genotypes at the peak markers were identified, and six such individuals were detected in the population. These individuals tended to show higher SPAD values than YSG1; however, the difference was not statistically significant (Figure 5).

### 3.4. Sequence Analysis of Candidate Genes

Candidate genes, *EF-G* and *GUF1*, were selected based on their putative functions related to chloroplast processes and photosynthesis. SNPs identified in these genes were synonymous substitutions, and no amino acid changes were detected (Supplementary Table S6).

### 3.5. Correlation Between Gene Expression and SPAD Values

No clear correlation was observed between transcript abundance and SPAD values for *EF-G* (*r* = 0.036) and *GUF1* (*r* = 0.006) in the $F_{2}$ population (Supplementary Figure S1).

## 4. Discussion

### 4.1. Validity of SPAD Values as a Trait Indicator

Leaf greenness is a complex trait determined by the combined effects of multiple pigment compounds. In contrast, SPAD values represent an optical index that can be measured objectively and conveniently, making them a useful indicator of leaf greenness.

In this study, $F_{1}$ and $F_{2}$ populations derived from a cross between the green cultivar KUJYO and the dark green cultivar YSG1 were used to measure SPAD values and pigment compounds, including chlorophyll *a*, chlorophyll *b*, β-carotene, and lutein. Broad-sense heritability was estimated to evaluate the genetic contribution to these traits.

The results showed that SPAD values exhibited high broad-sense heritability ($H^{2}=0.76$) and a continuous distribution in the $F_{2}$ population, indicating that this trait is quantitatively controlled by multiple genetic loci (Figure 1, Table 1). In contrast, pigment compounds showed broad-sense heritability values close to zero in this population, suggesting a strong influence of environmental factors and indicating that these traits are less suitable for genetic analysis (Supplementary Table S3).

SPAD values have been widely used as an indicator reflecting chlorophyll content, and previous studies have applied SPAD values as a target trait for QTL analysis [14,23]. Based on these findings, SPAD values, which are strongly influenced by genetic factors, were selected as the target trait for QTL analysis in this study.

### 4.2. Construction and Evaluation of the Linkage Map

Bunching onion is an outcrossing species with a high level of heterozygosity, and the development of inbred lines is therefore difficult [24]. Consequently, the number of markers showing homozygous genotypes between the parental lines (A and B) is limited. When linkage analysis of the $F_{2}$ population was performed using only these markers, the resulting linkage map showed low density with large gaps.

To address this limitation, in addition to markers showing homozygous genotypes in the parental lines (A × B), markers showing combinations of homozygous and heterozygous genotypes (A × H or H × B) were included in the linkage analysis. As a result, the density of the linkage map was improved, allowing broader genomic regions to be covered (Figure 2). However, the use of heterozygous markers requires the estimation of allelic phase, which increases the complexity of linkage analysis [25]. In fact, the initial linkage map constructed in this study exhibited a redundant structure. Therefore, marker selection criteria were made more stringent, and filtering based on missing data and segregation distortion was reinforced to improve the accuracy of the linkage map.

Nevertheless, discrepancies in marker order between the genetic linkage map and the physical map were observed in several genomic regions. The degree of discrepancy varied among QTL regions, with relatively good collinearity observed in the QTL3 region, whereas markers in QTL1, QTL2, and QTL4 were distributed across broader physical intervals. These discrepancies may be attributable to multiple factors, including uncertainties in phase estimation of heterozygous markers, variation in recombination rates, structural variations, and genetic divergence from the reference genome [26,27]. In addition, bunching onion has a large genome rich in repetitive sequences [12], which can hinder unique read mapping and lead to uncertainty in the estimation of physical positions. However, despite these limitations, clear LOD peaks were detected in the QTL analysis based on the linkage map constructed in this study (Figure 3), suggesting that the identified QTLs are associated with SPAD values. Further analyses using larger populations and fine mapping will be necessary to improve the resolution of these QTLs.

### 4.3. QTL and Candidate Genes

The QTL regions identified in this study contained genes encoding *EF-G* and *GUF1* (Table 3). These proteins are involved in chloroplast translation, with EF-G mediating ribosomal translocation during elongation and GUF1 (a homolog of LEPA) functioning as a regulatory factor that maintains translational efficiency and fidelity [28,29].

Photosystem II (PSII) is highly susceptible to damage under light stress, and its functional maintenance depends on protein synthesis in chloroplasts, including the resynthesis of the D1 protein. Impairment of EF-G function has been shown to affect chloroplast protein synthesis required for PSII repair [30], and disruption of chloroplast EF-G leads to reduced chlorophyll accumulation and impaired chloroplast development [28]. In addition, mutants of GUF1, a chloroplast translation factor, exhibit reduced photosynthetic activity and pale green phenotypes [29]. These observations suggest a link between chloroplast translation and chlorophyll accumulation.

However, comparison of the coding sequences of the candidate genes between the parental lines revealed no amino acid substitutions (Supplementary Table S6). Furthermore, no clear correlation was observed between transcript abundance and SPAD values in the $F_{2}$ population (Supplementary Figure S1). These results suggest that variation in SPAD values cannot be fully explained by differences in coding sequences or gene expression levels alone, indicating the possible involvement of regulatory mechanisms beyond the transcriptional level. Further studies will be required to clarify the functional involvement of these genes.

### 4.4. Implications for Marker-Assisted Selection

The parental line YSG1 exhibited higher SPAD values, whereas KUJYO-derived alleles increased SPAD values at some QTLs, indicating that favorable alleles are distributed between the parental lines (Table 3). Furthermore, the mean SPAD values of individuals carrying favorable allele combinations at QTL2, QTL3, and QTL4 were not significantly different from those of the high-SPAD parental line YSG1; however, their comparable phenotypes suggest that these allele combinations may be useful for improving leaf greenness. Given the limited sample size in this study, further validation using larger populations and diverse environmental conditions will be necessary to confirm the effectiveness and reliability of these markers.

Overall, the QTLs identified in this study provide a useful foundation for DNA marker development and may contribute to improving selection efficiency in breeding programs aimed at enhancing SPAD values.

## 5. Conclusions

This study revealed that SPAD variation in bunching onion is controlled by multiple genetic factors through QTL analysis. In particular, major QTLs detected on chromosomes 4 and 5 contained candidate genes related to chloroplast translation; however, no clear functional differences were observed at either the sequence or expression level. These results suggest that variation in SPAD values cannot be explained solely by differences in coding sequences or transcriptional levels, but may be regulated by more complex mechanisms. In contrast, individuals carrying combinations of favorable alleles contributing to increased SPAD values exhibited phenotypes comparable to the high-SPAD parent. These findings indicate that the QTLs identified in this study have potential utility for marker-assisted selection.

However, given the limitations in population size and environmental conditions, further validation using larger populations and more diverse conditions will be necessary to assess the stability and practical applicability of these findings.

Overall, this study provides a foundation for understanding the genetic regulation of leaf greenness in bunching onion and contributes to the development of molecular breeding strategies for improving this trait.

## Figures and Tables

**Table 1:** Summary statistics of SPAD values in the parental lines, $F_{1}$, and $F_{2}$ population.

Line	N	Mean ± SE	Variance	$P_{1}$ vs. $P_{2}$	Broad-Sense Heritability
KUJYO ($P_{1}$)	10	31.8 ± 0.56	3.17	***	–
YSG1 ($P_{2}$)	10	44.9 ± 0.69	4.74	–	–
$F_{1}$	10	38.0 ± 0.51	2.64	–	–
$F_{2}$	79	39.0 ± 0.43	14.46	–	0.76

*** indicates a significant difference between $P_{1}$ and $P_{2}$ at the 0.1% level (Student’s *t*-test).

**Table 2:** Summary of linkage groups and marker distribution.

	Chr1	Chr2	Chr3	Chr4	Chr5	Chr6	Chr7	Chr8	Total
Total Length (cM)	304.9	210.1	283.6	385.0	282.8	326.1	162.2	148.3	2103.0
No. of markers	144	149	97	72	80	104	67	52	765

**Table 3:** Summary of QTLs associated with SPAD values identified in the $F_{2}$ population. For each QTL, the chromosome (Chr), peak position (cM), peak marker, logarithm of odds (LOD) score, percentage of phenotypic variance explained (PVE), additive effect, 1.5-LOD support interval (cM), corresponding physical interval (Mb), significance level, and candidate genes are shown.

QTL	Chr	Peak (cM)	Peak Marker	LOD	PVE (%)	Additive	1.5-LOD Support Interval (cM)	Physical Interval (Mb)	Significance	Candidate Genes
QTL1	4	36.14	–	8.12	38.1	+3.82	20.14–50.14	–	1%	–
QTL2	4	63.67	AlfiFF_005592.p1	7.91	37.3	+2.83	57.67–71.45	1133.8	1%	Translation factor GUF1 homolog, chloroplastic
QTL3	4	201.69	AlfiFF_034125.p1	5.51	27.8	−1.59	194.66–208.01	827.6–837.9	1%	Uncharacterized proteins
QTL4	5	172.52	AlfiFF_022605.p1	5.42	27.4	−2.45	145.61–177.35	114.8–636.5	5%	Elongation factor G
										PI4 kinase
										UDP-galactose transporter
										transcription-related proteins

## Data Availability

The data are available from the corresponding author upon reasonable request.

## Supplementary Materials

The following supporting information can be downloaded at: https://www.mdpi.com/article/10.3390/genes17050534/s1. Figure S1. Relationship between transcript abundance and SPAD values for (A) *EF-G* and (B) *GUF1* in the $F_{2}$ population. Transcript abundance was expressed as $\log_{2}$(CPM + 1). Pearson’s correlation coefficients (*r*) are indicated in each panel. Table S1. Meteorological Data for the Cultivation Site from 12-May to 19-July, 2021. Table S2. SPAD values and pigment contents in parental lines (Kujyo ($P_{1}$) and YSG1 ($P_{2}$)), $F_{1}$, and $F_{2}$ populations. Missing data are indicated as NA. Table S3. Summary statistics of pigment compounds in the parental lines, $F_{1}$, and $F_{2}$ population. Table S4. List of SNP markers used for linkage map construction. Table S5. Markers located in QTL regions for SPAD values, including marker names, corresponding full IDs (Allium TDB), chromosome assignments, genetic positions (cM), and physical positions (Mb). Markers included are located within the 1.5-LOD support interval and flanking regions of each QTL. Table S6. Codon substitutions between KUJYO and YSG1 in candidate genes located within QTL regions.
