# Supplementary material for: QTL Mapping of SPAD Values Associated with Leaf Color in Bunching Onion
Source: Genes (Basel). 2026 Apr 30;17(5):534. doi: 10.3390/genes17050534 (PMC13206149; doi:10.3390/genes17050534)
Supplement: Supplementary file 1 [file genes-17-00534-s001.zip › QTL_ FigureS1.pdf]

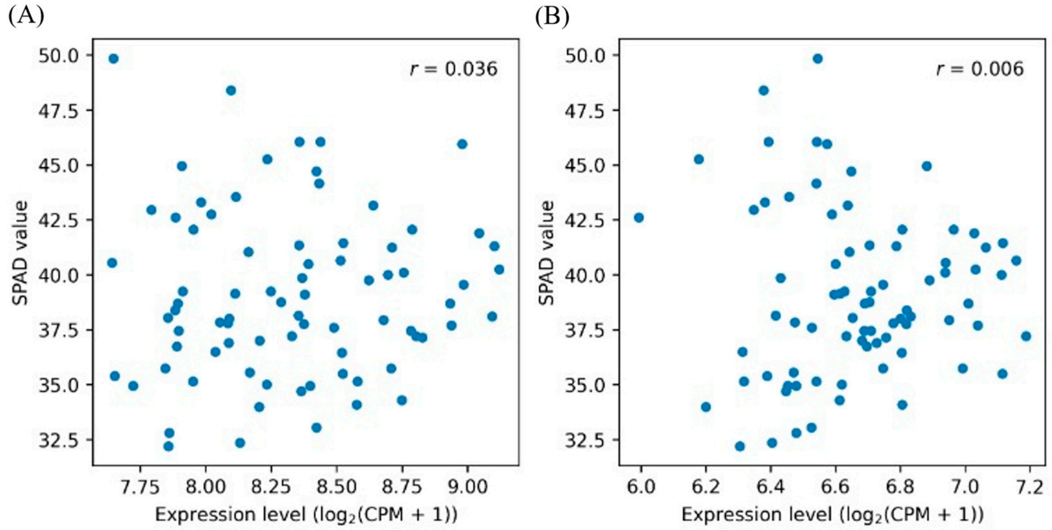

Figure S1. Relationship between transcript abundance and SPAD values for (A) *EF-G* and (B) *GUF1* in the F_2 population. Transcript abundance was expressed as $\log_2(\mathrm{CPM} + 1)$. Pearson's correlation coefficients (*r*) are indicated in each panel.
